# Social agents as catalysts: Social dynamics in the classroom with book introduction robot

**DOI:** 10.3389/frobt.2022.934325

**Published:** 2022-11-23

**Authors:** Hirotaka Osawa, Kohei Horino, Takuya Sato

**Affiliations:** ^1^ Human-Agent Interaction Laboratory, Faculty of Science and Technology, Keio University, Yokohama, Kanagawa, Japan; ^2^ Human-Agent Interaction Laboratory, Graduate School of Science and Technology, University of Tsukuba, Tsukuba, Ibaraki, Japan

**Keywords:** human-agent interaction, active learning, human-robot interaction, human-interface design, robotics

## Abstract

One of the possible benefits of robot-mediated education is the effect of the robot becoming a catalyst between people and facilitating learning. In this study, the authors focused on an asynchronous active learning method mediated by robots. Active learning is believed to help students continue learning and develop the ability to think independently. Therefore, the authors improved the UGA (User Generated Agent) system that we have created for long-term active learning in COVID-19 to create an environment where children introduce books to each other *via* robots. The authors installed the robot in an elementary school and conducted an experiment lasting more than a year. As a result, it was confirmed that the robot could continue to be used without getting bored even over a long period of time. They also analyzed how the children created the contents by analyzing the contents that had a particularly high number of views. In particular, the authors observed changes in children’s behavior, such as spontaneous advertising activities, guidance from upperclassmen to lowerclassmen, collaboration with multiple people, and increased interest in technology, even under conditions where the new coronavirus was spreading and children’s social interaction was inhibited.

## 1 Introduction

Currently, the educational environment is seriously understaffed. In order to help children’s education, it is important to help motivate not only teachers but also children themselves to actively learn. Active learning has been proposed as one method to improve this situation. Compared to conventional knowledge acquisition learning, in which learners watch lectures, active learning is a more active learning process. Active learning has been reported to be particularly effective in STEM fields (Science, Technology, Engineering, and Mathematics) (Freeman et al., 2014). It is also expected to develop general abilities including cognitive, ethical, social, cultural, knowledge, and experience skills through learners’ self-motivated engagement in learning. Active learning is considered to be useful for cultivating the ability to continue learning throughout life and the ability to think independently, and is important for responding to the rapidly changing society. As teaching materials for active learning, applications in the form of drills in which students answer questions presented on a tablet terminal, or in the form of graphical programming in which students carry out missions, have been proposed. Such applications have the advantage that users can proceed with learning by themselves without a teacher. On the other hand, the scope of learning is limited to a predefined range.

The other method involves the intervention of a robot or agent to amplify the educational effect. Many educational robots are currently being developed to assist in these situations, and it is hoped that their physicality can be used to develop the cognitive and emotional abilities of the educated (Belpaeme et al., 2018).

We have attempted to combine the advantages of active learning and social robots, and tried a method to promote spontaneous learning, i.e., active learning, by learners using a robot that operates asynchronously. We have proposed a UGA (User Generated Agent) system as a method to motivate learners to expand the scope of their own learning (Kudo et al., 2016), (Sato et al., 2017). UGA is a programming material in which a speech program for introducing books is created on an application, and the robot controls speech, body movements, and facial expression changes. By providing content created by learners to other learners, a wide variety of content is updated in a fluid manner, and learners who view the content are motivated to read the book that was the subject of the introduction. The users act on each other to expand the learning range of the other, thereby achieving a spontaneous expansion of the learning range.

In this study, UGA was implemented as a robot, and the effectiveness of the UGA method was evaluated by operating it so that it would function smoothly over a long period of time. Feedback was provided mainly by checking the number of views on the content creation screen, but if children could directly evaluate the quality of their content, they would be able to obtain direct feedback on their own content. Therefore, this study analyzed how spontaneous learning is generated from children’s behavior and content.

## 2 Related research

There have been several attempts to use robots to support learning. In this section, we discuss these related studies and explain how our asynchronous content method contributes to the situation.

### 2.1 Method in which the robot itself becomes a teaching aid

In the case of the educational introduction of the robot agent, it is important to determine how the robot’s role is set up. Belpaeme et al. (2018) categorize the role of autonomous robots in education as teacher, peer, and novice. (Kanda et al. (2004) used two Robovie robots to help first- and sixth-grade Japanese students learn English for 2 weeks. The robots acted as peer tutors and called the learners’ names. The learners interacted with the robot frequently during the first week. However, the number of interactions decreased rapidly in the second week.

This study focused on the role of the robot as a first-time learner in motivating children out of their social roles. Tanaka and Matsuzoe. (2012) used a robot that incorporated the learning by teaching method to support english learning in Japanese 3- to 6-year-old children. The robot was designed to support Japanese 3- to 6-year-old children in learning English by using a learning by teaching robot. The robot was set at a lower learning level than the learner and was in a position to receive instruction from the learner. By stimulating the learner’s motivation to care for the robot, we were able to encourage spontaneous learning in order to teach the robot.

### 2.2 Methodology of combining an educational application and a robot

In this type of educational robot, the learner faces the learning material together with the robot and receives support from the robot. In the early stages of learning, the robot learned as well as the learner, and the percentage of correct answers was about the same as the learner. However, as the learning progresses, the robot becomes more efficient and improves its response rate. By observing the robot’s efficient learning, the learner is expected to acquire similar learning skills. As a result, learners imitated the robot’s learning method and learned the same effective learning method as the robot. Aditi et al. combined NAO and a tablet device to support fifth and sixth graders in math learning (Ramachandran et al., 2019). The robot was linked to a hint function implemented in a learning application on a tablet device. When the learner continuously gets incorrect answers without using a hint, the robot gives a hint on its own. When the learner continuously asks for a hint, the robot refuses to give a hint and encourages the learner to try first. As a result, the learner refrains from using the hints without asking for them, and the learning method becomes one in which the learner tries first, which also improves the learning outcome.

### 2.3 User generated agent: A method that encourages mutual learning among learners

In this research, the robot is positioned not as an autonomous entity but as an avatar that acts on behalf of children. The robot acts as an intermediary to help children learn and create by creating an environment in which children can teach each other. By encouraging mutual learning among children in the form of the robot teaching the learner the contents of the book, the learner can indirectly benefit from the learning. By using the robot as an intermediary, the robot can support learning by generating user-to-user communication, such as teaching each other between children in different grade levels or collaborative work by multiple users.

Compared to similar methods, this method is unique in that it focuses on communication between children, and asynchronous communication is generated through content created by the children, rather than direct contact between them. We believe that this point especially becomes an advantage of continuous operation under COVID-19.

## 3 Design of user generated agents

A user-generated agent is a system that applies user-generated content (UGC) to an agent system, which is not content created by professionals like news and newspapers, but by ordinary people. UGC has the characteristics of introducing unique viewpoints of users and unstable quality of contents (Ramachandran et al., 2019). Compared UGC and non-UGC, and found that UGC has features such as significantly higher production speed, easy creation of content by anyone, and user participation in the form of feedback through comments and favorite functions.

UGA is a combination of UGC and robot programming; in addition to the advantages of traditional user-generated content (UGC), UGA allows users to have interaction motivation due to the agent nature of anthropomorphic agents (Kudo et al., 2016), (Sato et al., 2017). Fogg. (2002) pointed out that anthropomorphic agent systems create long-term trusting relationships with users, and such long-term relationships with agents may encourage agents to listen to content and create agent content.

As in the previous study (Sato et al., 2017), we used an elementary school curriculum book introduction as the content subject in this study. Book introductions have the effect of increasing the reading motivation of people who are introduced to books by introducing impressive scenes from the books. On the other hand, UGC is characterized by the objective evaluation from the user’s point of view, which leads to a sense of familiarity and is more effective than advertising by a company, and we believe that there is a high affinity between book introductions and UGC.

In the book introduction, children create an introduction of a book they find interesting and want others to read, and give a speech about it. In UGA, students insert commands to control the robot’s movements in the same introduction and create a program to do the speech. Active learning includes the following elements: deciding how to use the system independently, deciding what books to introduce, trial-and-error in creating the introduction and agent, and obtaining feedback. Figure 2 shows the interaction model of UGC and UGA, in which the agent acts as a mediator between users’ interactions. The active learning elements include deciding to use the system voluntarily, deciding which books to introduce, trial-and-error in creating introductions and agents, and obtaining feedback.

**FIGURE 1:**
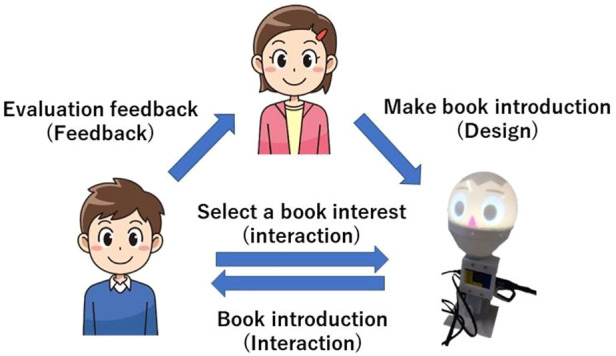
Interaction in user generated agent.

In this research, we aim to promote children’s interest in books by creating cycles of Design, Interaction, Feedback. The proposed interaction model is shown in Figure 1. That cycle has a dual effect. One is the effect of receiving book introductions from agent, and the other is the effect of considering introduction contents.

Details of the proposed interaction model are shown below.

### 3.1 Book introduction agent

**FIGURE 2:**
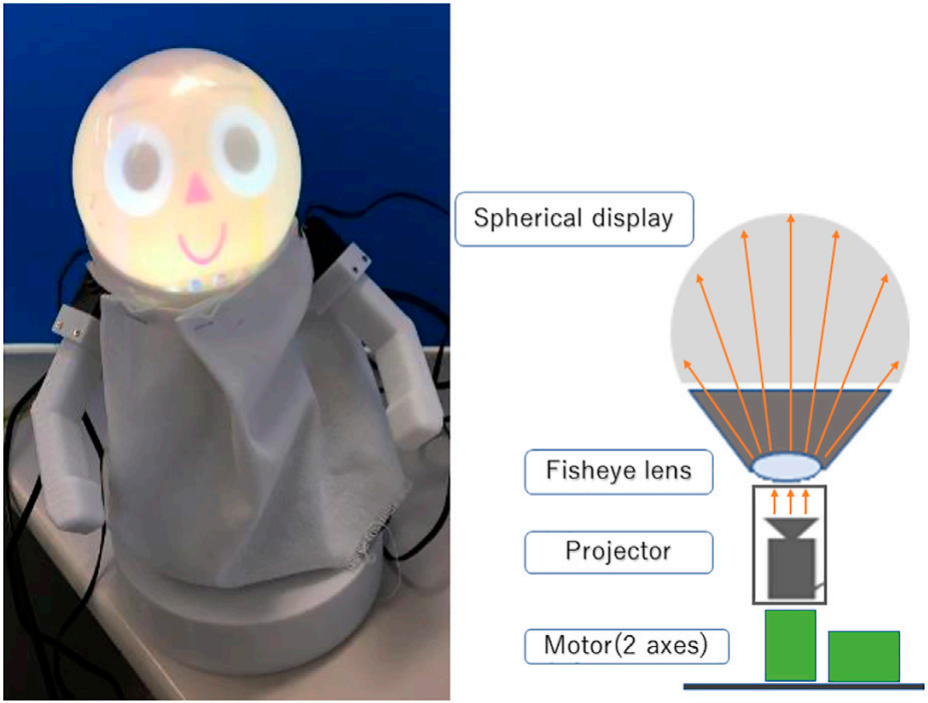
Appearance of UGA (left) and illustration of face (right).

In this study, we use a social agent that allows flexible representation by children. Figure 2 shows the details of the robot’s face. The system consists of an agent creation screen, contents list screen, feedback screen, face viewer, and output destination robot. Each program is connected *via* TCP communication. The created contents are stored in. csv files and can be accessed at any time. The data that can be retrieved from the software are the created content data, the creation date and time of the content, the number of times the content has been played, and the number of times the content has been rated highly. A list of all contents and a time-stamped history are recorded separately, and the two are cross-checked to ensure data consistency. The robot is able to.

1) Express nonverbal expressions through body movements
2) Express non-verbal expression through changes in facial expressions

**FIGURE 3:**
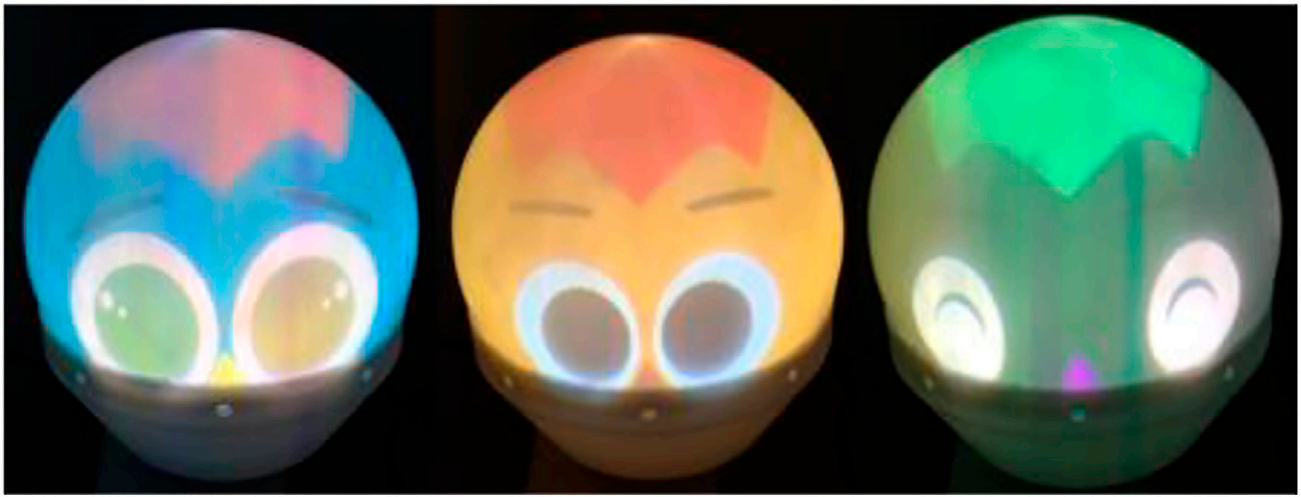
Color examples of UGA.

**FIGURE 4:**
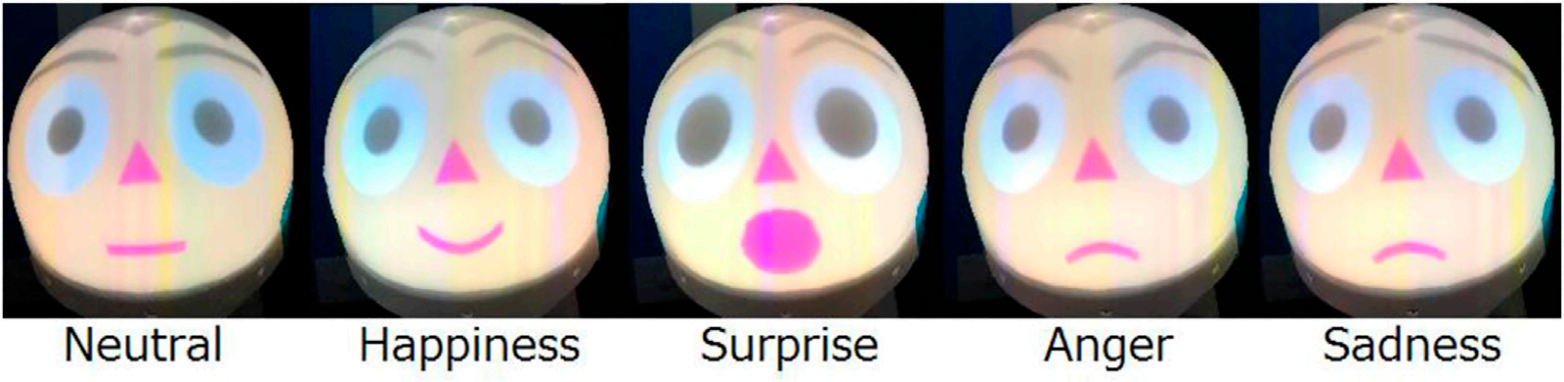
Examples of emotion of robot.

Two servo motors are installed in the robot. These motors allow the face to move at any angle in the longitudinal and transverse directions. The robot’s face is attached to an upper spherical surface. The projector is installed inside the robot. The facial expressions of the robot occur faster than those of a conventional mechanical face robot because the projector allows facial expressions at 60 fps. It is also possible to create various facial expressions by changing the shape, size, color, eyes, nose, and mouth. Figure 3 shows examples of facial expressions. For the skin and hair colors, in addition to the skintones by Fitzpatrick, we added colors that could not be possible in reality, which could reflect elements within the story such as fantasy (Levenson et al., 1995). Figure 4 shows examples of the emotions. The agent shows an interest in the children by displaying the neutral emotion and four basic emotions of six emotions proposed by Ekman (Ekman and O’Sullivan, 1979). Fear and Disgust is difficult to recognize by user so we removed them.

To realize the expression of nonverbal expressions through body movements, the robot is equipped with two arms and a waist joint. In addition, a total of four servo motors are incorporated in each part of the robot: one for vertical arm motion, one for forward leaning motion, and one for left-right turning motion. The servo motors are KONDO KRS-3204 ICS. The parts were manufactured using a 3D printer and made of polycarbonate.

Screws and nuts are used to connect the parts. A mobile projector and a spherical display were combined to form the head in order to realize the expression of nonverbal expressions through changes in facial expressions. The head is tilted forward to increase the face area when viewed from the front. The mobile projector is a SK Telecom SmartBeam. The agent drawing screen contains the face drawing process, synthetic voice output process, and motor control process necessary for content playback. After receiving content, the agent reads the plain text portion of the speech and executes inserted commands as needed. The face rendering process is implemented in OpenGL, and can be adjusted by editing an external configuration file so that it is rendered correctly in accordance with the projection environment. It can express normal, smiling, confused, startled, and angry expressions. The synthesized voice output process is implemented with the Microsoft Speech API, and the reading speed and pitch of the voice can be specified by the content side.

### 3.2 Designer application

**FIGURE 5:**
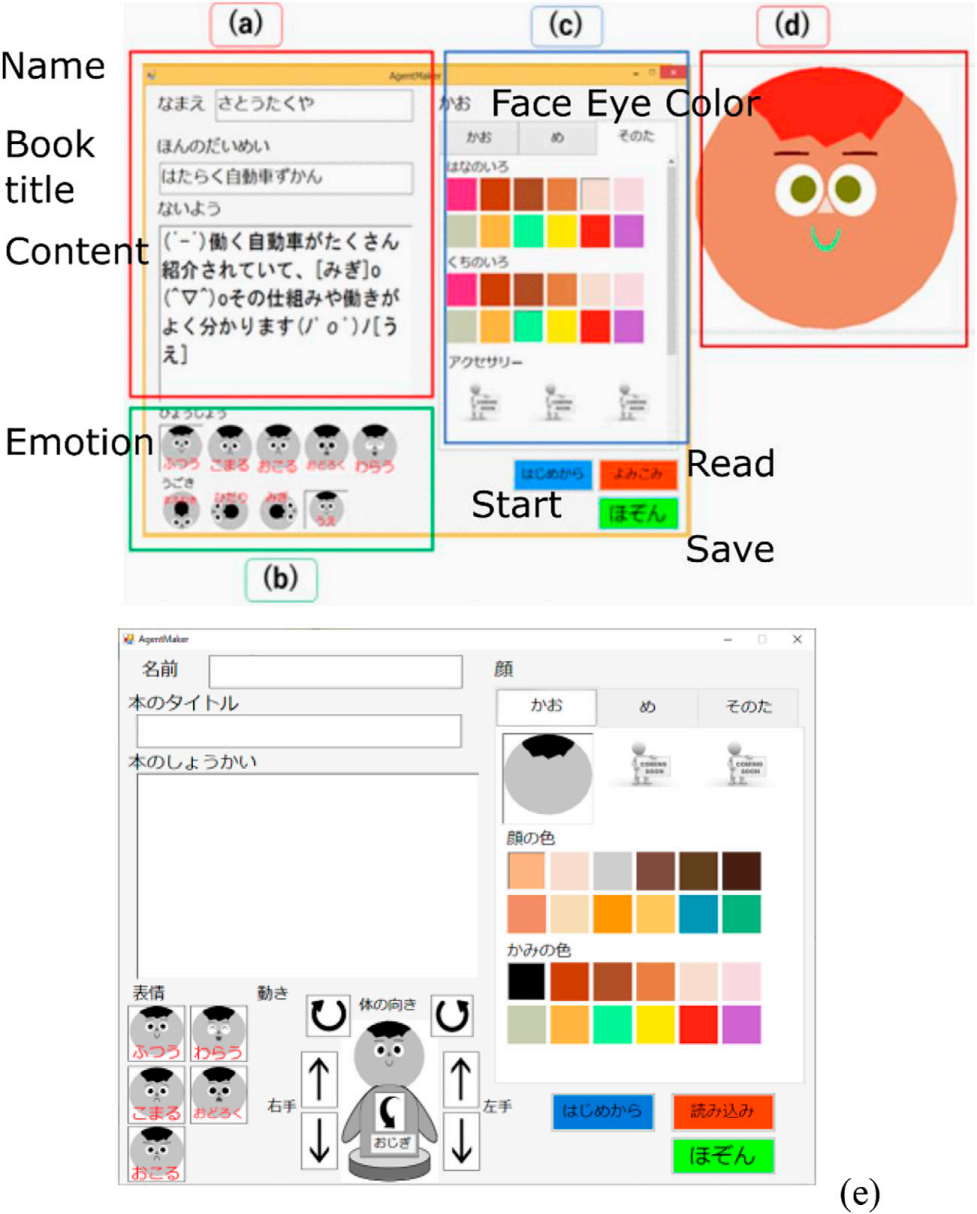
Designer application. **(A)** Name, title, and content of the book the user wants to introduce. **(B)** Expression and movement of the robot. **(C)** Robot’s facial parts, skin color, hairstyle, hair color, eye, and nose size and color. **(D)** Preview. **(E)** Body movement.

We also created a design application for the children. With the UGA designer, children themselves can design the size and color of facial parts, facial expressions, and the robot’s utterance content. Figure 5 shows the application screen. As for the appearance of the agent, the appearance of the agent can be changed to match the book the user wants to introduce, allowing it to reflect the user’s creativity in the same way as user-generated content. Among the anthropomorphic expressions, the facial expressions and tone of voice, which are likely to contribute to the agent’s expressive judgment, were the targets that can be changed by the child. The agent is designed from an interface placed on the right half of the screen. The appearance of the agent is designed by selecting the shape and color of the hair, eyes, nose, and mouth. The voice can be adjusted in three levels: fast (+10), normal (±0), and slow (−10), and pitch in three levels: high (+4), normal (±0), and low (−4). Each value was determined from the Microsoft Speech API specification. Users can not only create new content, but also read and improve previously created content.

The forms (A)–(E) shown in Figure 5 each have the following functions:

A) The user can enter the name, title, and contents of the book that he/she wants to introduce.
B) The user can change the expression and movement of the robot in the content by buttons.
C) The user can change the face parts of the robot. They are the skin color, hairstyle, hair color, eyes and nose size, and color.
D) A preview of the face created in form (c) is illustrated.
E) Updated application for adding body movement.

Users can create content by entering the necessary information on this screen. The content consists of four elements: 1. The creator’s name and group name, 2. The title of the book to be introduced, 3. The speech text, and 4. The agent’s design. Speech sentences are created by arbitrarily inserting commands to control the robot’s facial expressions and body movements into plain text. Commands can be inserted simply by clicking on the buttons below the speech text input area. Each button has a simple indication of the action to be performed by the robot according to the command.

### 3.3 Feedback

To achieve the purpose of the UGC detailed in Section 2.1, it is o feedback the behavior of the user to the creator. We constructed a system that automatically generates feedback. We measure the number of times the introduction was listened to, the number of times the book was picked up, and the number of times the book was borrowed, and feed them back to the creator. The details of the sensing processes are shown below.

#### 3.3.1 Measurement of the children’s interest

**FIGURE 6:**
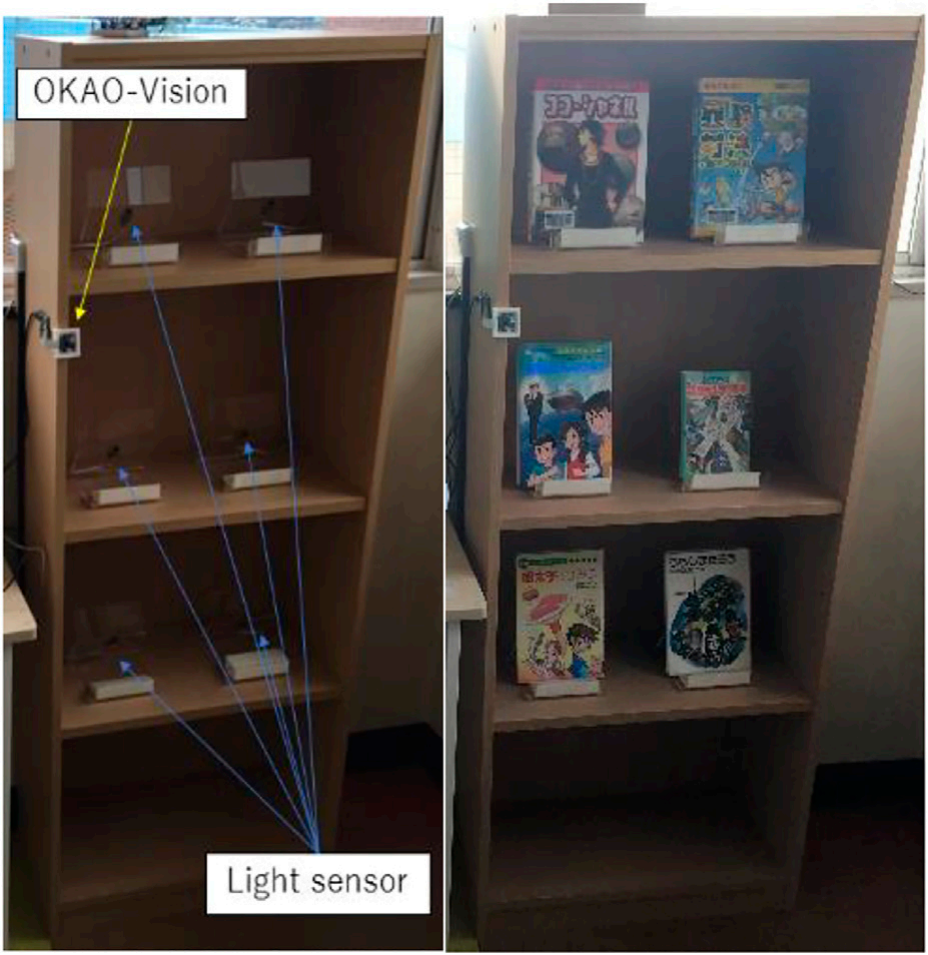
Bookshelf with sensors installed, as shown on the left: OKAO-VISION detects the location of the face, and the light sensor detects the presence of the book. The right side shows the situation where the book is placed.

We measured whether children pick up a book following the book introduction. Light sensors were installed in the holder on the bookshelf to judge whether a book was picked up. We displayed the front covers of the books in order to make it easier to understand the books the robot introduces. The facial sensor OKAO-Vision was installed in order to measure whether the user’s gaze was guided to the book by the robot. Figure 6 shows the installed bookshelf.

#### 3.3.2 Feedback application

**FIGURE 7:**
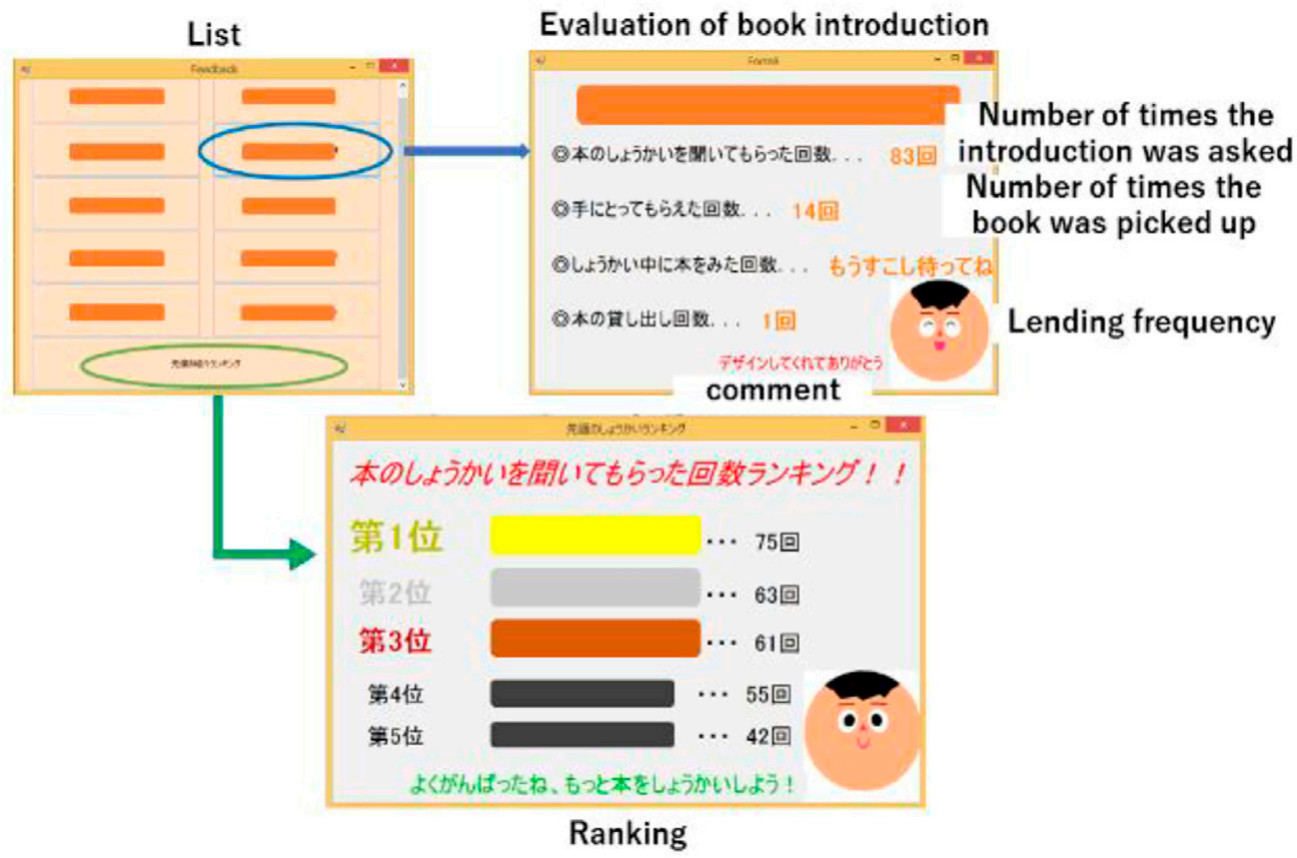
Feedback application. The upper left corner shows the number of times the book was introduced, picked up, or checked out. The lower figure shows the ranking of the number of book introductions viewed.

We developed a feedback application that gives feedback to children who created book introductions in the UGA system. Figure 7 shows the application screen. The book title is shown in boxes on the list screen, although these boxes are hidden to protect privacy.

**FIGURE 8:**
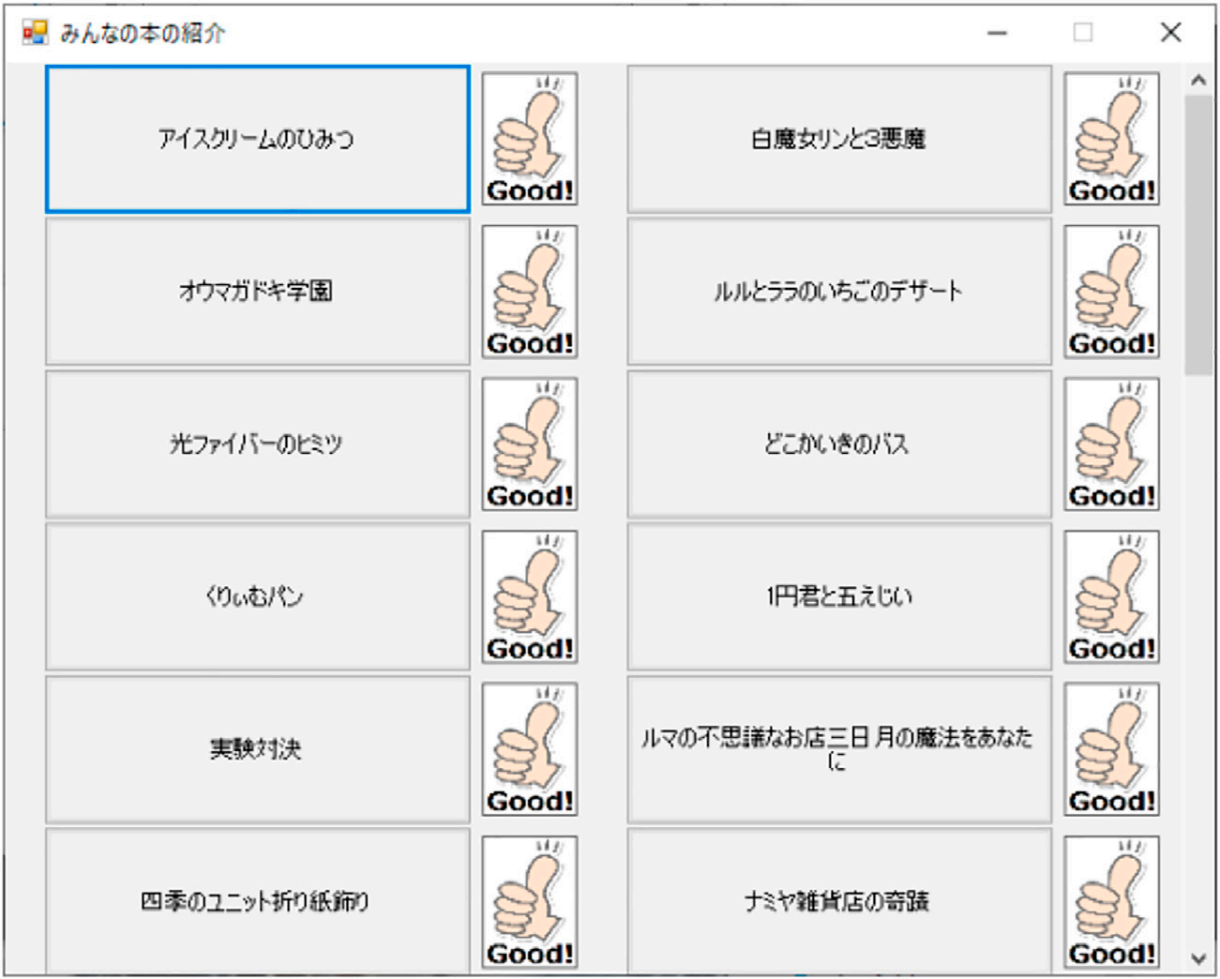
Screen for returning ratings during the feedback application. Children who listen to the content introduction press the good button if they liked the introduction content of the book.

Figure 8 shows screenshot of feedback application. The content playback screen lists content created by the user, and by clicking on the content, data is sent to the robot, which then begins to play the content. After content playback, users who feel that the content is of high quality can send feedback to the content creator by clicking the high evaluation button located to the right of the play button.

## 4 Evaluation of UGA

For evaluation, a field experiment was conducted with the cooperation of Imakashima Elementary School in Tsukuba City. The experiment period was 490 days (288 days of actual operation) from 21 June 2019 to 23 October 2020. The experiment was conducted under COVID-19 from April 2020 onward. The participants of the experiment are all children enrolled in the school who visit the library and wish to use the UGA. There are 10–20 children present in each grade level. No rewards will be given to the subjects. The subjects were highly motivated to use information terminals, such as using smartphones on a daily basis. The older students receive instruction in PC-related technologies in class, and are fully proficient in mouse and keyboard operations necessary for content creation.

The robot are located in a corner of the library. A PC for content creation, a PC for robot control, an agent robot, and a web camera for observation are installed as a set. Each PC is connected *via* mobile Wifi and can be remotely controlled from a university PC *via* VPN. The agent robot is fixed to the table and faces the front when it is not playing contents.

During the 233rd day of operation from the start of the experiment under COVID-19, a workshop was held for eight children who wished to “get to know the robot better,” in which we explained the operation of the robot, taking care of its hygiene from the problems related to COVID-19. This was due to the fact that they tried to use the device alone and gave up soon after using it for a short time, and that they had to close applications running on their experimental PCs in order to use them for watching videos and playing games, which was a major burden on the operation of the device. The librarian of the library reported that the causes of the problem were as follows: 1. The number of participants in the experiment had changed over the school year, 2. The children did not understand the reason why the robot was here, 3. The children misunderstood that the PC could be used for play, and 4. The number of children who did not know what to do increased. The report stated that the number of children who do not know what to do has increased. Based on this, we prepared a detailed manual. 6.

## 5 Results of the evaluation experiment

**FIGURE 9:**
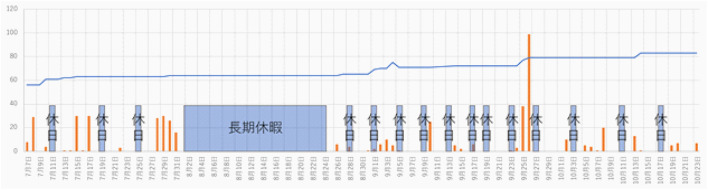
Short blue areas show holidays and long blue area shows summer vacation.

Figure 9 shows the changes in the total number of contents from 7 June 2020 to 23 October 2020, as well as the number of plays per day. The line graph shows the total number of contents, and the bar graph shows the number of times the contents were used each day. From 08/01 to 08/24, there is no change in the total number of contents because of the summer vacation period, and the number of times the system is used is 0. The total number of contents decreased on September 03 due to the censorship and deletion of problematic contents; on September 26, the contents were replayed one by one from the beginning during a short lunch break, confirming a phenomenon in which the number of replayed contents was prominent. During periods when no contents are added, the number of usage may drop to 0. However, when contents are added, it is evident that the contents are used again.

### 5.1 Results for the workshop

When we attempted to paste the manuals in the workshop, we received the following comments from the children who participated in the workshop.

1) They would like to visit each class with the manual and explain about the UGA
2) Want to know more about the robot (technology). 3.
3) To be able to fix the robot by themselves when it stops
4) Want to name the robot

The participants offered to name the robot. The place where they were put up was on the wall in front of the experimental apparatus.

**FIGURE 10:**
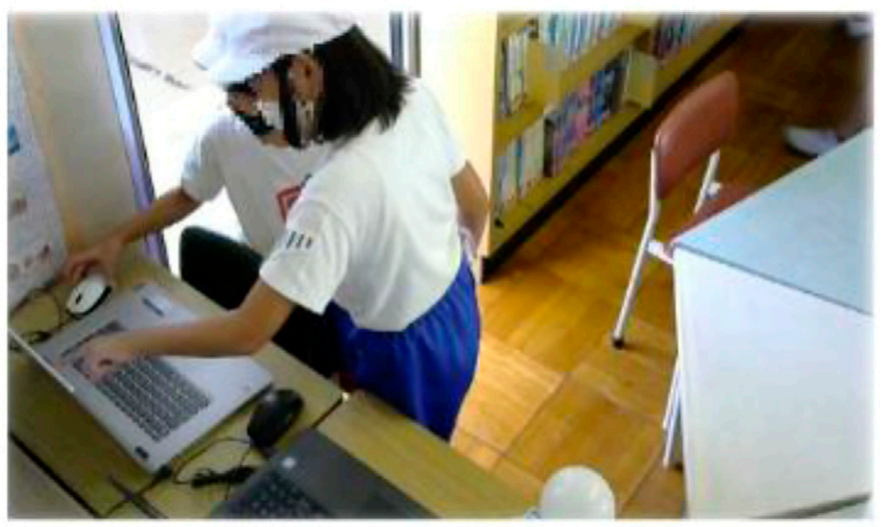
Instruction from a senior classmate and a junior classmate.

Figure 10 shows one child using the robot on another child. An experienced senior student is assisting a junior student, who is using the device for the first time, in the creation of contents. At this time, the student gives specific instructions by pointing to the screen.

In addition, many children gathered in the library during the lunch break, and the following were observed.

1) Consultations among several students, such as exchanging ideas, for example, “It would be nice if the last command was (laugh).
2) “Next one, please. You’ll do it, right? (a statement that is considerate of other users) .
3) Comments that are considerate of other users, such as “00 (title of the book) .... It's interesting, is not it? I think we should do it, too.” Statements that indicate an increased willingness to use the system among children who have not yet used it. 4.
4) Communication across the grade levels, such as an experienced senior student accompanying a junior student who is new to the system

The occurrence of communication across grade levels, such as an older student who is familiar with the system accompanying a younger student who is new to it, was confirmed.

## 6 Discussion

### 6.1 Considerations on the ability to solve the problem of boredom

In the previous results, the UGA system stopped updating its contents after 54 days of operation, indicating that the system had become bored. The current results show that the UGA system has been in continuous use for more than a year without being completely bored, as it was used more than 10 times a week in the last 2 months and did not stop creating content, on average, two times a week.

We believe that the workshop was an opportunity for active children to expand the community by naming robots, conducting robot maintenance activities, and visiting classrooms to advertise UGA to children who have not yet joined the user community. The increase in the number of users due to the expansion of the community is considered to be effective in solving the problem of boredom when UGA stops updating its contents by increasing the speed of content updating and the diversity of its contents. In addition, an increase in the number of participants will have a beneficial effect on the acquisition of social skills, as more people will engage in co-creation activities. UGA is considered to be effective in solving the problem of boredom caused by long-term use of the system.

It has been confirmed that the UGA system creates a learning ecosystem in which children teach each other. In this context, there is an occurrence of UGA-specific communication across grade levels, such as upperclassmen instructing lowerclassmen. The promotional activities conducted this time by visiting each class are considered to be similar to this unique type of communication.

### 6.2 Consideration of the workshop

Due to the influence of COVID-19, the workshop was attended only by users who strongly requested to participate. We believe that by providing them with the opportunity of the manual, some obstacles that they could not clear by themselves were removed, and they started to improve the environment spontaneously. The ability to plan and execute, such as taking a school-wide survey to decide on a name for the robot. Spontaneous learning, such as proactively asking questions about the mechanisms and hardware technology for maintenance and operation. Dissemination of information, such as classroom visits to advertise UGA’s existence and what it can do. If these actions can be induced by reaching out to people who are interested in the system, they will contribute to achieving the expectations of active learning, which is the development of generic competencies including cognitive, ethical, social, cultural, knowledge, and experience competencies through learners’ spontaneous engagement in learning.

### 6.3 Considerations for the creation of a learning ecosystem

In the created contents, 40% of the contents changed the hair color from the default black, 58% changed the eye size from normal, 22% changed the eye color from the default black, 41% changed the skin color, 30% changed the flower color, and 19% changed the voice speed. 19% changed the speed of the voice. Many of the subjects changed the color of their eyes and skin when the subject matter was fantasy, which is far from reality, or when the subject matter was horror. There were also examples of changing the manner of explanation to match the content, such as slowing down the speech in the case of horror.

**FIGURE 11:**
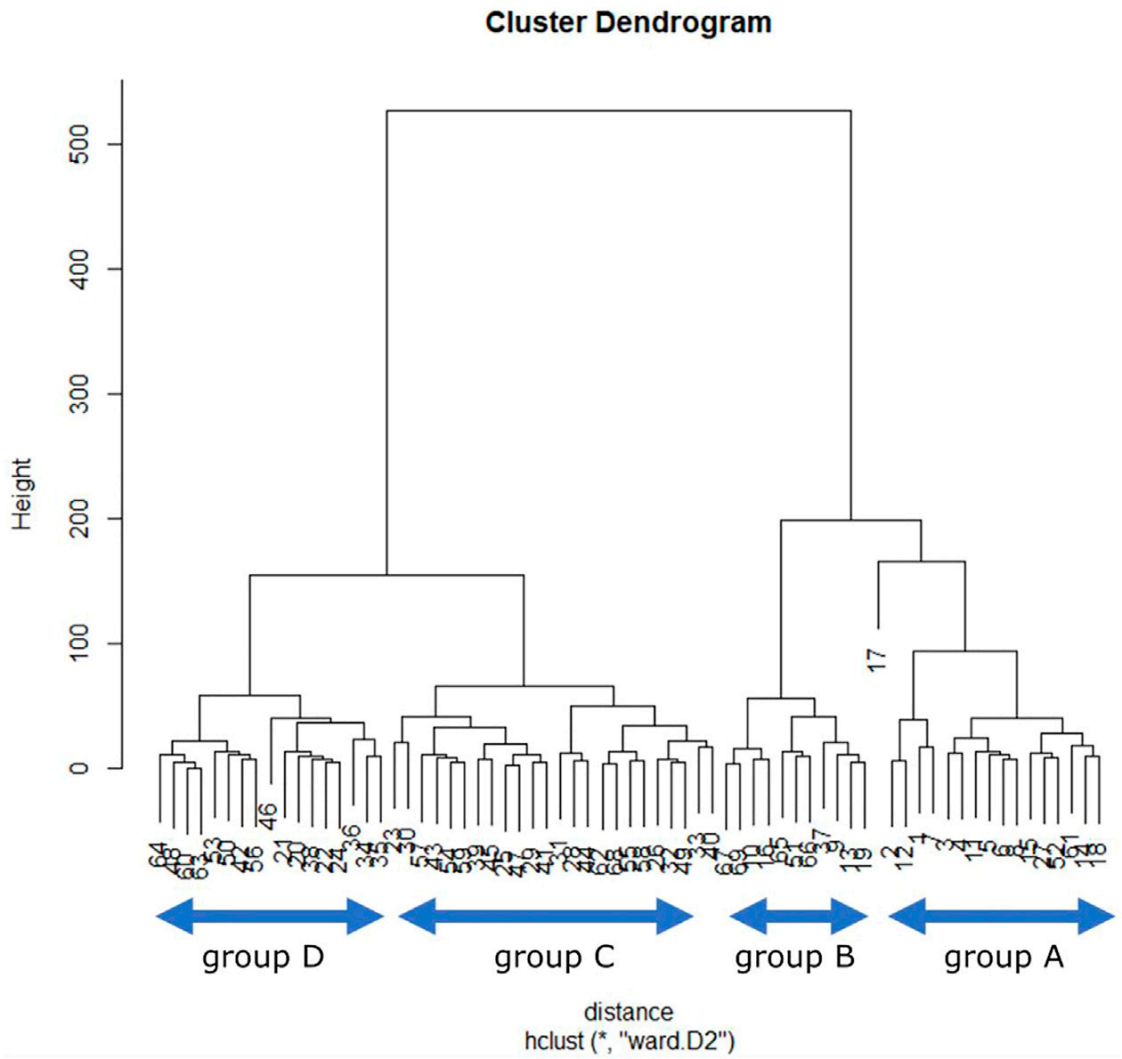
Hierarchical clustering results for 76 contents for book introduction.

The 76 created contents were analyzed by clustering. The items used were: 1. The number of characters; 2. The total number of facial expression changes; 3. The number of times each facial expression changed (smile, angry, confused, surprised, normal); 4. The total number of body movements; 5. The number of times each body movement was performed (right arm, left arm, bow, turn). Figure 11 shows the results of hierarchical clustering using the Ward method. In this study, we divided the samples into four groups: A, B, C, and D. Each group was characterized by the fact that the content in Group A was mostly well explained about the book, while Group D had particularly short content.

**FIGURE 12:**
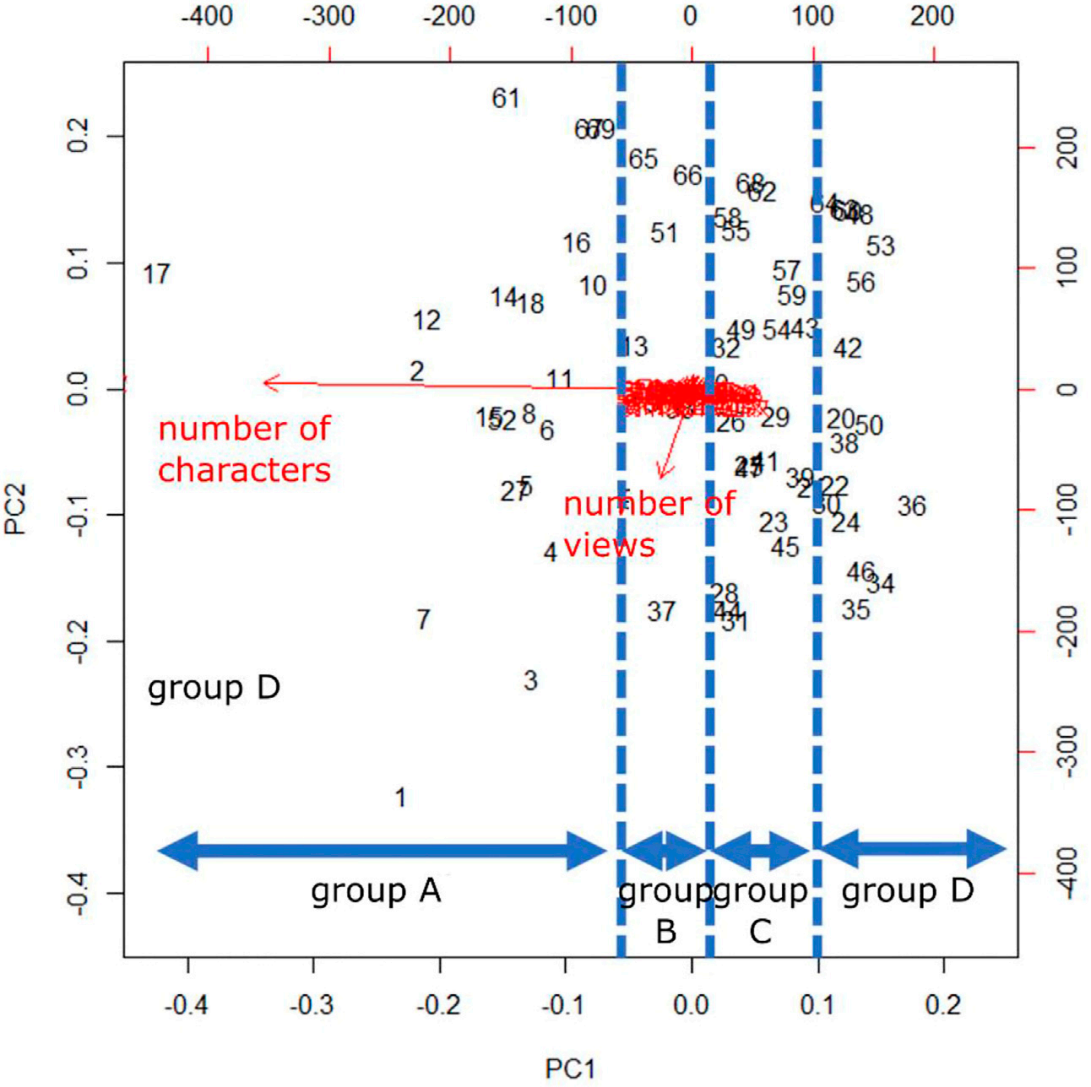
Results of principal component analysis for 76 content related to book introductions.

Figure 12 shows the results of principal component analysis. Each number in Figure 11 is in the order of content creation updates. It can be seen that the contents that had been updated tend to be younger in number and more attractive. A, B, C, and D in the figure correspond to the groups divided by the Ward method, respectively. PC1 is the number of characters, with a contribution ratio of 0.9412, and PC2 is the number of views, with a contribution ratio of 0.04324. This indicates that the number of characters and the number of views are characteristic factors. The main factor that separates the groups is the number of characters in the content. The average Good rate for each group was 0.190 for group A, 0.141 for group B, 0.074 for group C, and 0.065 for group D. It can be seen that content with a larger number of characters tends to have a higher Good rate. In addition, the number of views itself is also slightly higher for content with more characters.

Next, we analyze the contents of the individual contents, and find that the contents in group A have the following characteristics: 1.

1) Content that begins with a synopsis of the book, such as “This is a book about ________.”
2) Includes appealing points that the user finds interesting, such as “I like scene _____.”
3) Use many (smiles) and (turns) at the end of sentences
4) Use (fall forward) at the beginning and end of the content
5) Use (falling forward) at the beginning and end of the content.

The following are examples of content in Group A. This book is very interesting and full of stories. It's a very interesting book, even though it’s haunted (smiles). The title “Oumagadoki” does not mean that the horses are thrilled. (It’s about the time when you meet ghosts and demons, at dusk. Sounds interesting (smile), please read it. (turn) (turn) (fall forward).

Children tended to rate contents with a large number of characters highly. In particular, many of the contents in Group A have a structure that can be called a “beginning, middle and end.” It is thought that contents that not only have a large number of words, but also have a good structure and are of high quality as contents are highly evaluated. In addition, many of the contents are presented with (falling forward) at the beginning and end of the contents. This was thought to indicate that the children themselves had found a way to use the content as a form of greeting in presentations such as speeches. In this study, the physical movements performed by the robot were only indicated by arrows on the button for inserting body movement control commands on the content creation software. In contrast, we believe that the users found the meaning of the commands by seeing the actual movements of the robot. This suggests that, by leaving the judgment of meaning to the user, rather than presenting the meaning of the actions from the system designer’s side, it is possible to encourage the user to consider how to express what can be expressed by using the system, thereby encouraging the creation of knowledge.

This is the reason why we believe that the system can promote the creation of knowledge. From the above, we believe that feedback triggered the updating of content, which in turn generated attractive content. From this, we believe that UGA was able to create a learning ecosystem in which children teach each other. In addition, communication occurred in such a way that the ecosystem was maintained and activated.

## 7 Contributions and limitations

We conducted a 490-day field experiment using UGA in an actual elementary school. Since the participants in the experiment were elementary school students, problems occurred on average once or twice a day with system outages. This may have reduced the motivation of the participants who wanted to use the system but were unable to do so. In addition, during the experiment, the robot was frequently grabbed by hand and stopped while in motion. Although countermeasures against the danger of contact were taken at the design stage, active stopping of the robot was not considered. In addition, changes in the behavior of each age group within the elementary school, such as touching the robot because of interest or stopping the robot after thinking about it, were not considered, and may have an impact on the behavior of the children. This study was conducted only as a field experiment in an elementary school. The condition of the elementary school under COVID-19 is different from that under normal conditions, which may have an impact on the results.

## 8 Conclusion and future prospects

In this study, we conducted a 490-day field test using the proposed system and observed the behavior of children. We improved the operation method and constructed an environment that can maintain the learning ecosystem by children. In the previous study, the system was not used for 54 days after the start of operation due to boredom, but the system was used for 288 days in actual operation time. It was also confirmed that children spontaneously made requests to the author, such as “I want to do ____” and “I want to be able to do ____”. We believe that the communication between upper grade students and lower grade students, which was confirmed in the previous study, has developed into communication outside the school and across generations. The communication occurred on a large scale and transcended generations, not only among students but also among adults, such as: taking a school-wide survey to name the robot, actively asking questions about the mechanism and hardware technology for maintenance and operation, and visiting class rooms to advertise the existence of UGA and what it can do. Communication occurred on a large scale and across generations, not only among children, but also among adults. If these actions can be triggered by engaging with people who are interested in the system, it will contribute to the realization of a system that can achieve the expected results of active learning: the development of general abilities, including cognitive, ethical, social, cultural, knowledge, and experience skills, through learners’ spontaneous engagement in learning. UGA is currently being used by users on the system side. Currently, UGA does not have a function to call out to users from the system side. However, we believe that interaction with some active children and announcements of new contents will have a positive effect. For example, the robot calling out to the user by name is an effective interaction (Ramachandran et al., 2019), and including a function such as thanking the user when new content is added is expected to gain further acceptance and have a positive impact on the ecosystem. We would like to make every effort to realize a use that goes beyond simple learning support.

## Data Availability

The original contributions presented in the study are included in the article/Supplementary Material, further inquiries can be directed to the corresponding author.
